# Supplementary material for: Integrin α7 and Extracellular Matrix Laminin 211 Interaction Promotes Proliferation of Acute Myeloid Leukemia Cells and Is Associated with Granulocytic Sarcoma
Source: Cancers (Basel). 2020 Feb 5;12(2):363. doi: 10.3390/cancers12020363 (PMC7072541; doi:10.3390/cancers12020363)

PL21 p-ERK Laminin211

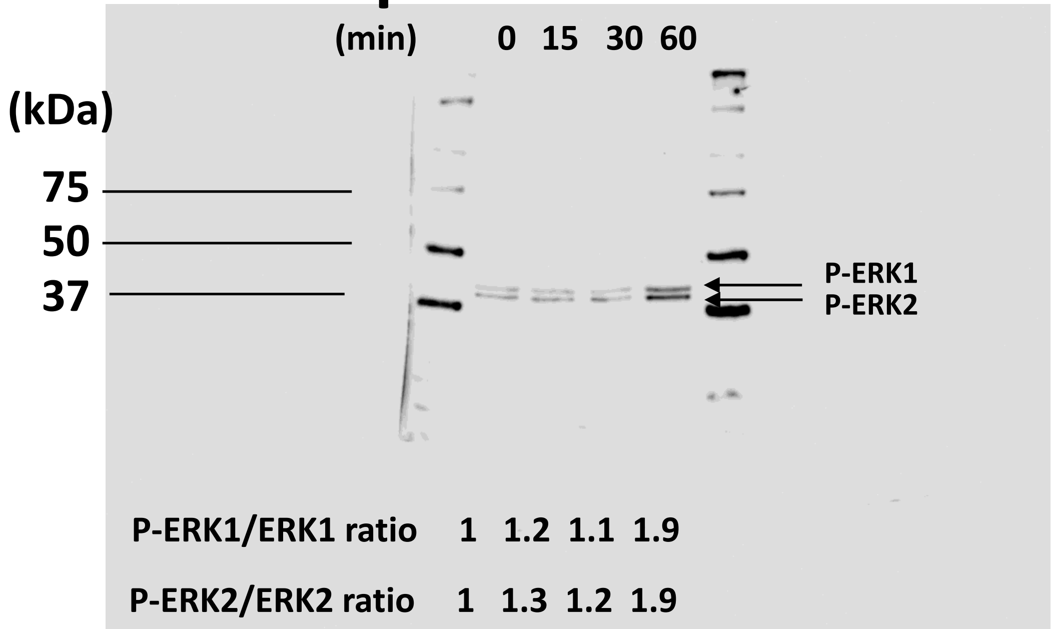

PL21 ERK Laminin211

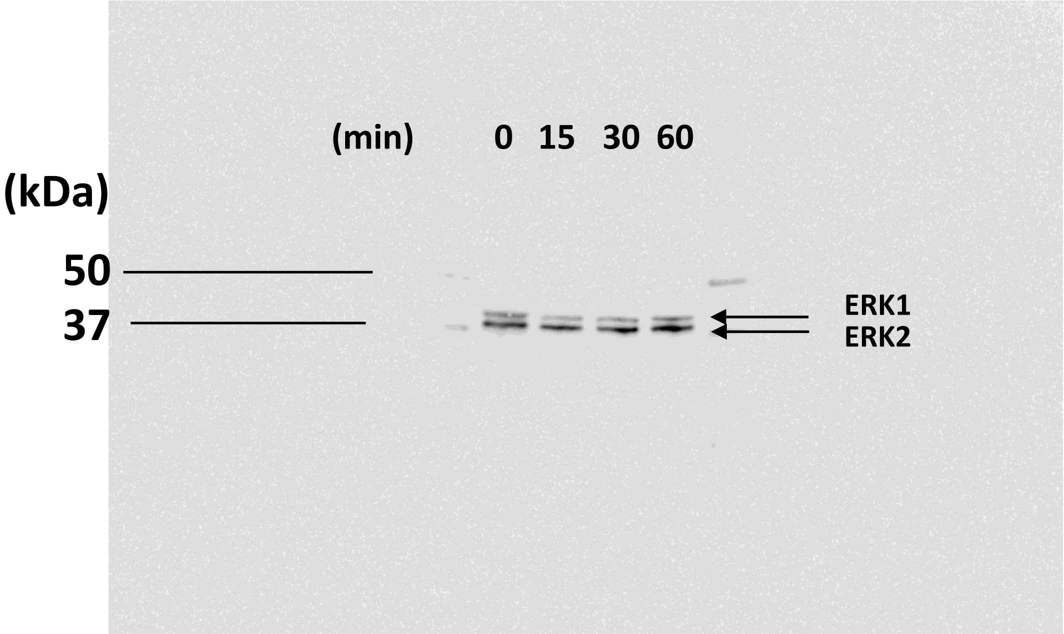

PL21 p-ERK Laminin411

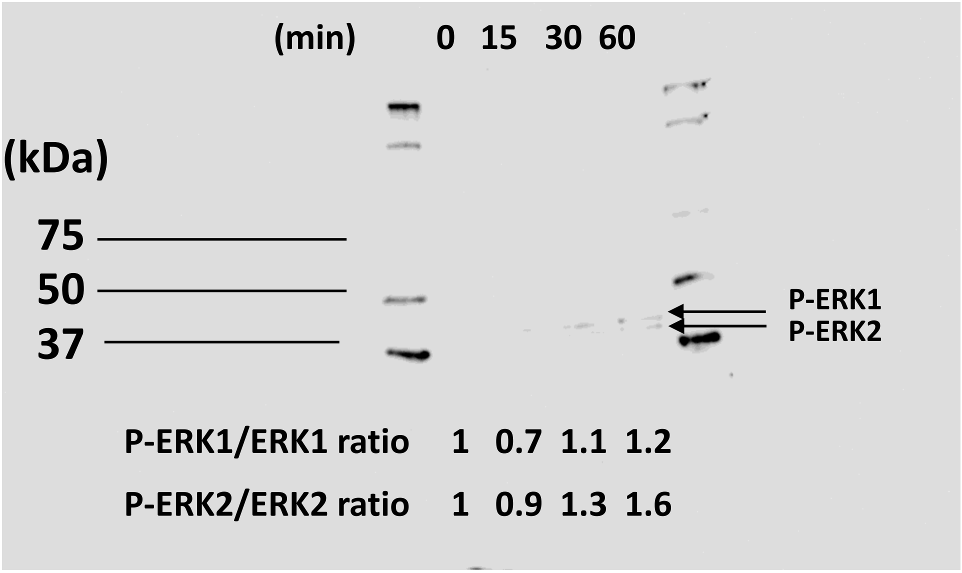

PL21 ERK Laminin411

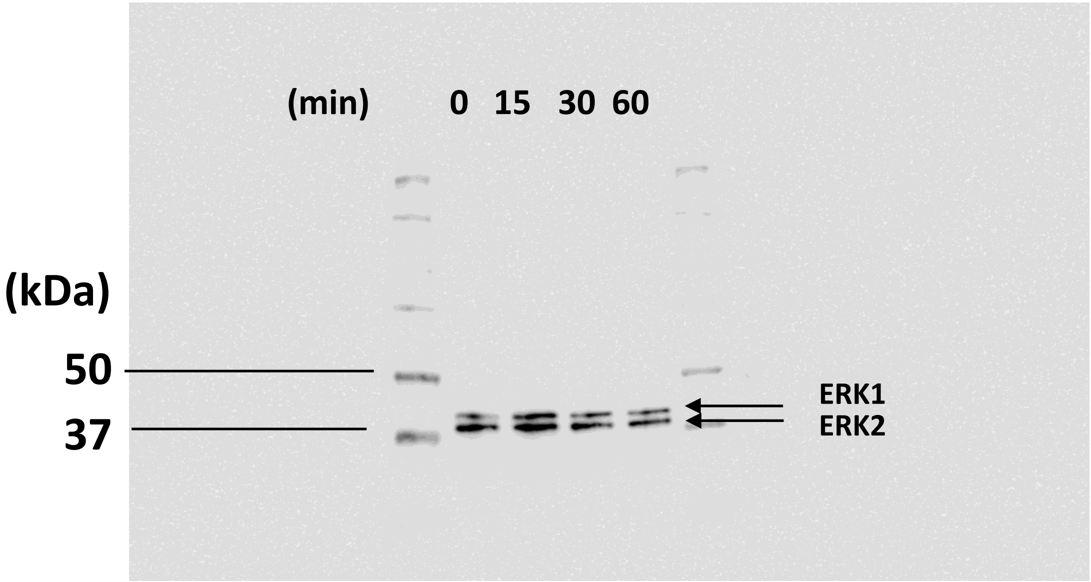

THP1 p-ERK Laminin211

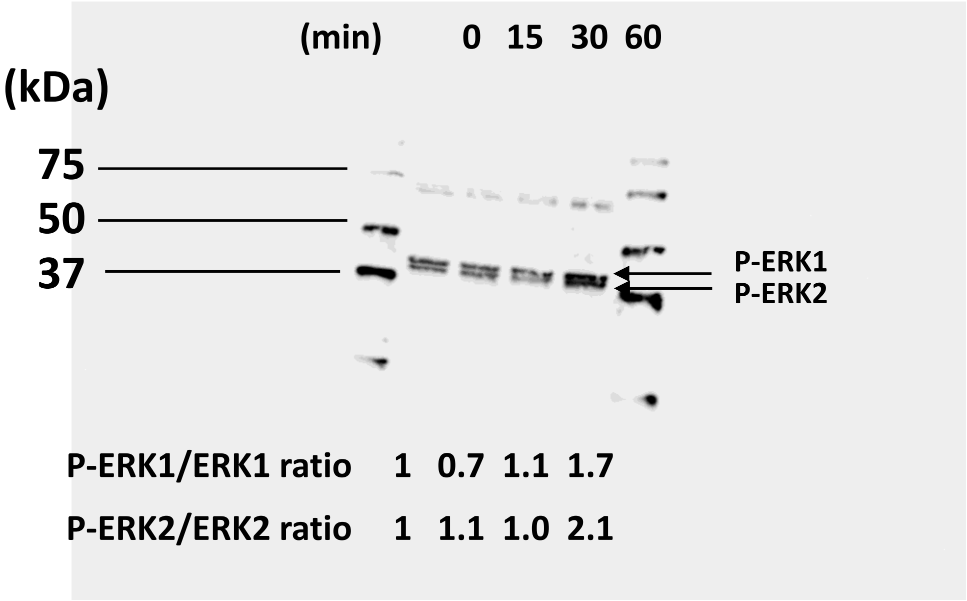

THP1 ERK Laminin211

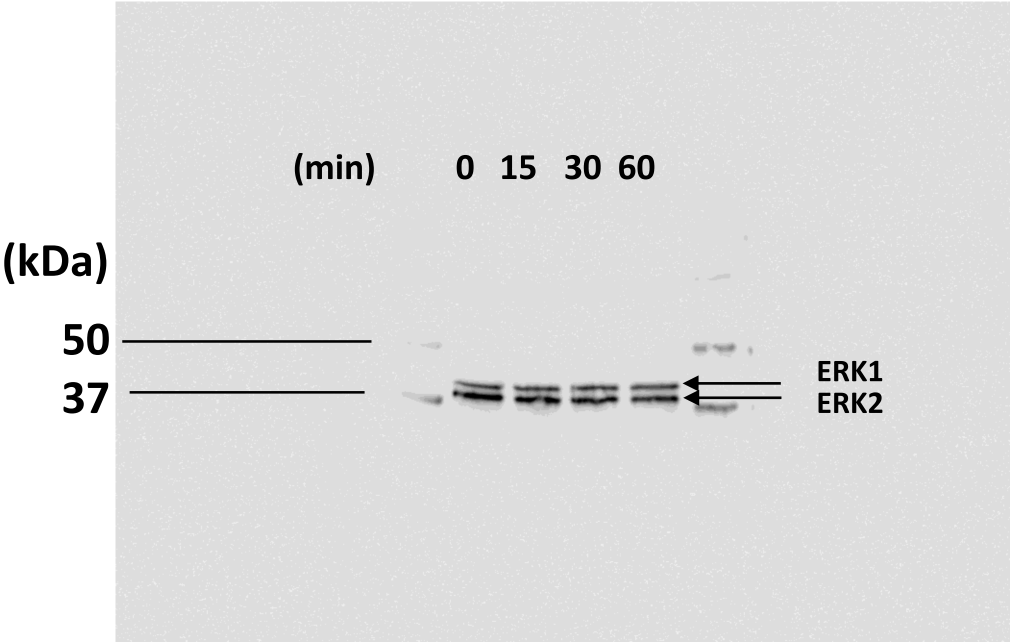

THP1 p-ERK Laminin411

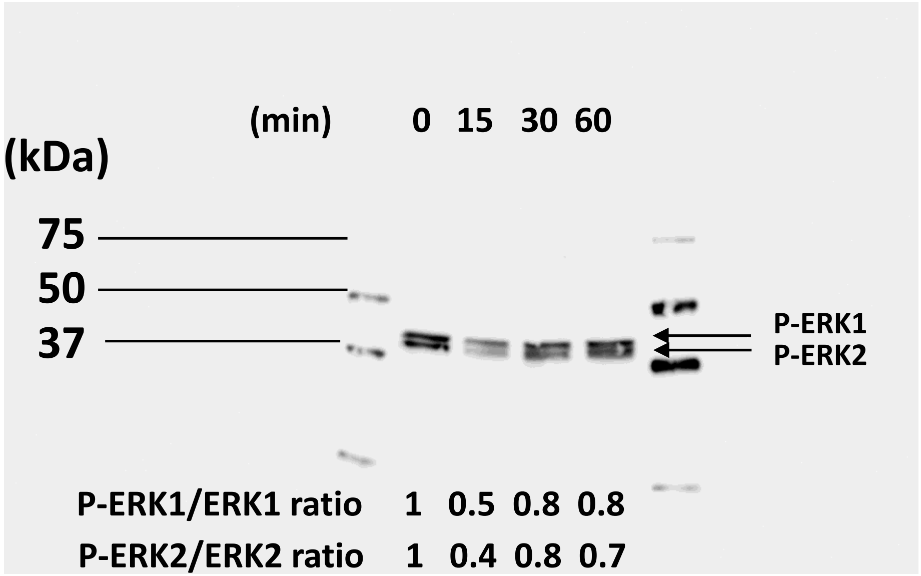

THP1 ERK Laminin411

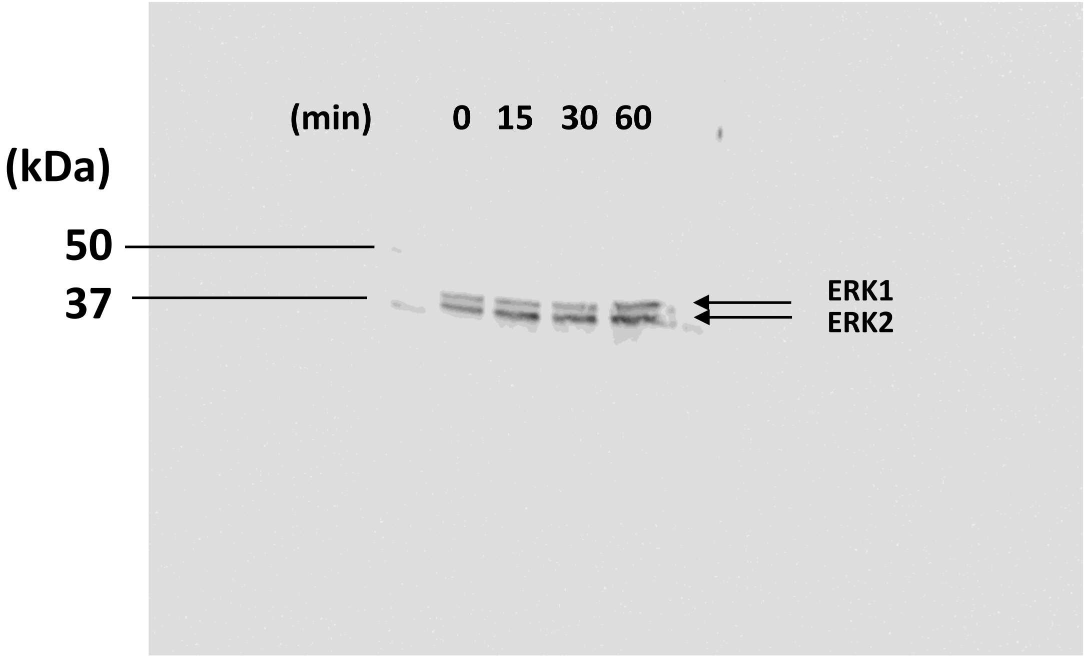

HL60 p-ERK Laminin211

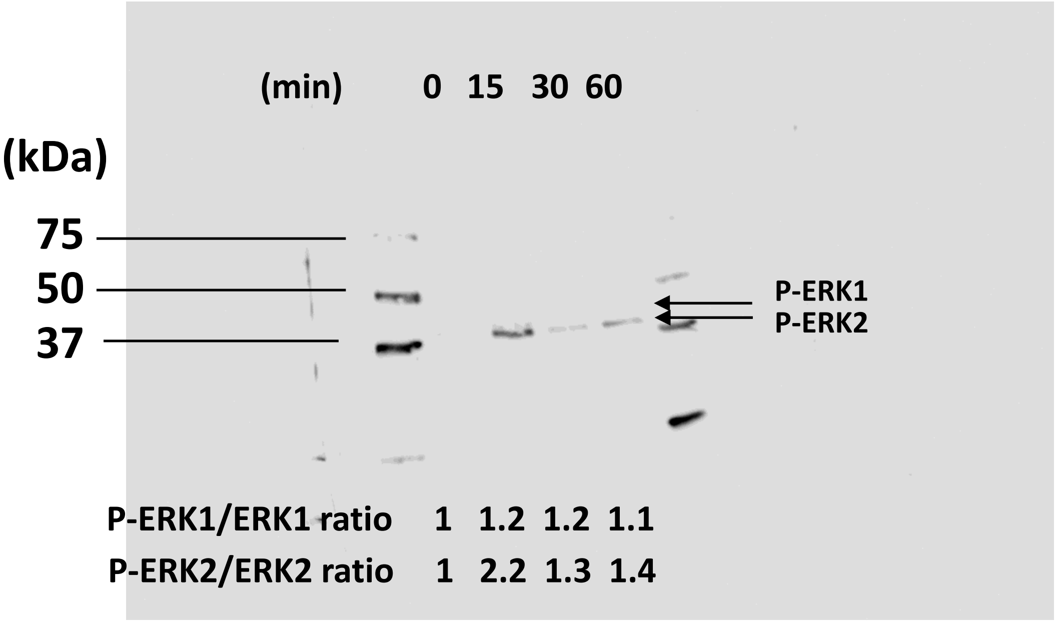

HL60 ERK Laminin211

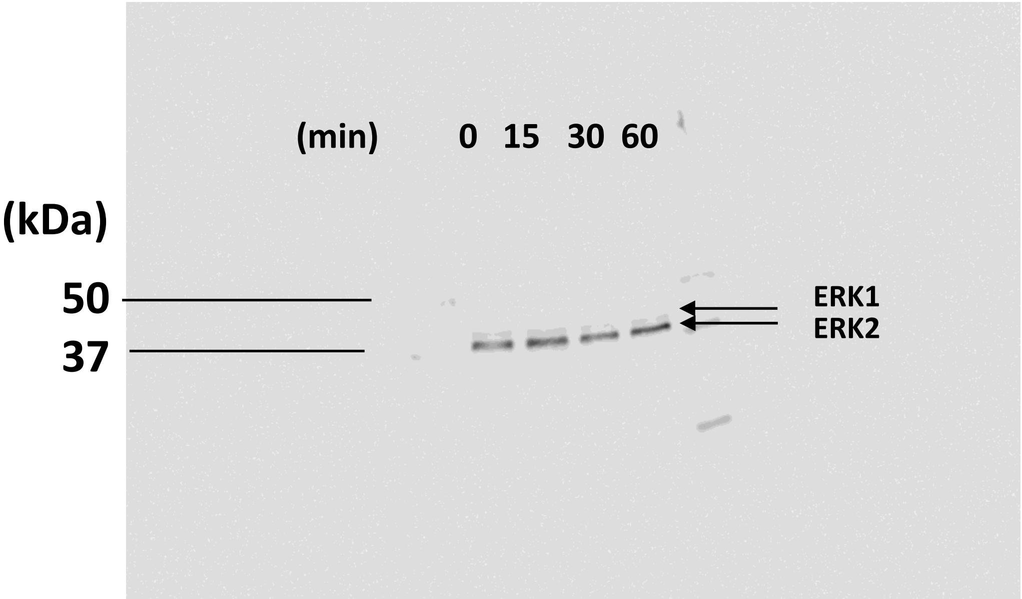

HL60 p-ERK Laminin411

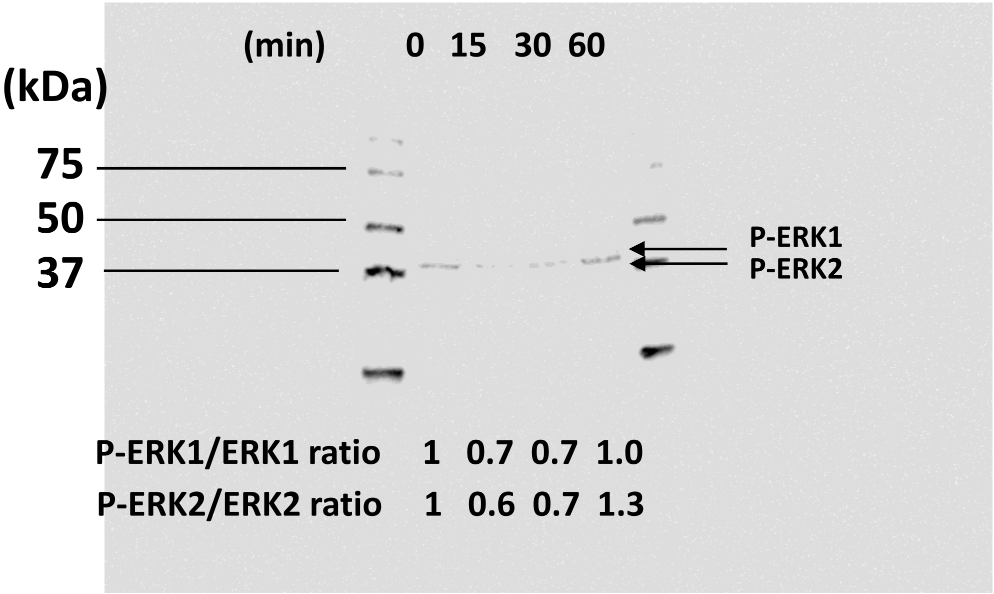

HL60 ERK Laminin411

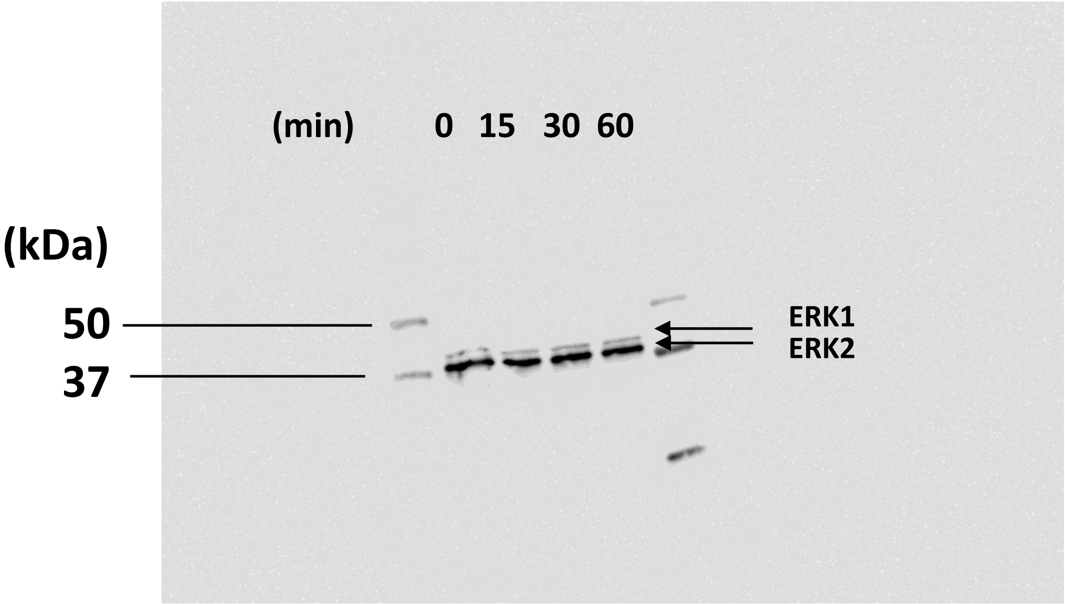

PL21 ITGA7 Knockdown

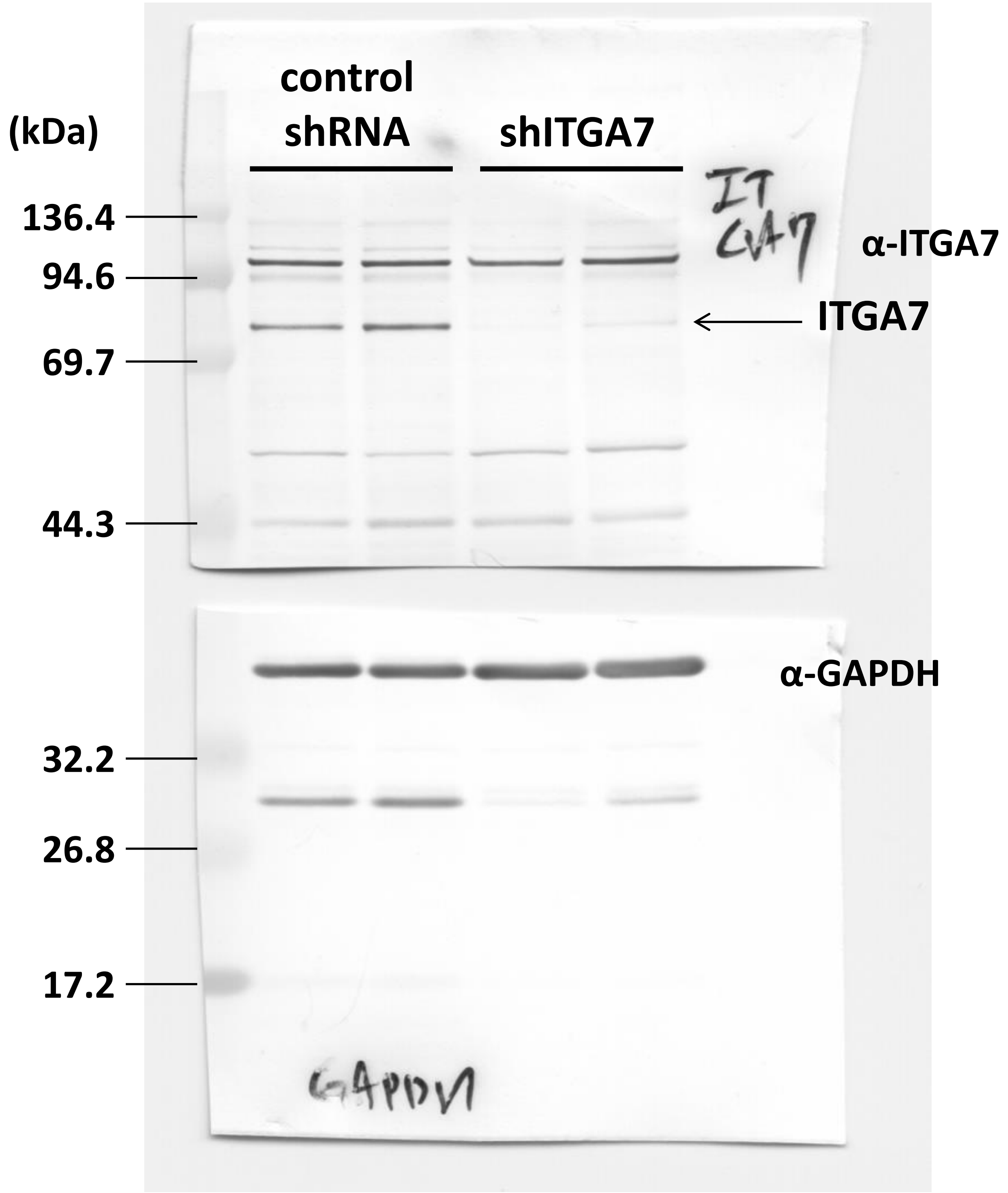

Supplement: Supplementary file 1 [file cancers-12-00363-s001.zip › Original Western Blot pictures.pdf]
