# Supplementary material for: Integrin α7 and Extracellular Matrix Laminin 211 Interaction Promotes Proliferation of Acute Myeloid Leukemia Cells and Is Associated with Granulocytic Sarcoma
Source: Cancers (Basel). 2020 Feb 5;12(2):363. doi: 10.3390/cancers12020363 (PMC7072541; doi:10.3390/cancers12020363)
Supplement: Supplementary file 1 [file cancers-12-00363-s001.zip › Supplementary Materials.pdf]

cancers

Supplementary Materials

Integrin α7 and Extracellular Matrix Laminin 211 Interaction Promotes Proliferation of Acute Myeloid Leukemia Cells and Is Associated with Granulocytic Sarcoma

Nobuhiko Kobayashi, Tsukasa Oda, Makiko Takizawa, Takuma Ishizaki, Norifumi Tsukamoto, Akihiko Yokohama, Hisashi Takei, Takayuki Saitoh, Hiroaki Shimizu, Kazuki Honma, Kei Kimura-Masuda, Yuko Kuroda, Rei Ishihara, Yuki Murakami, Hirokazu Murakami and Hiroshi Handa

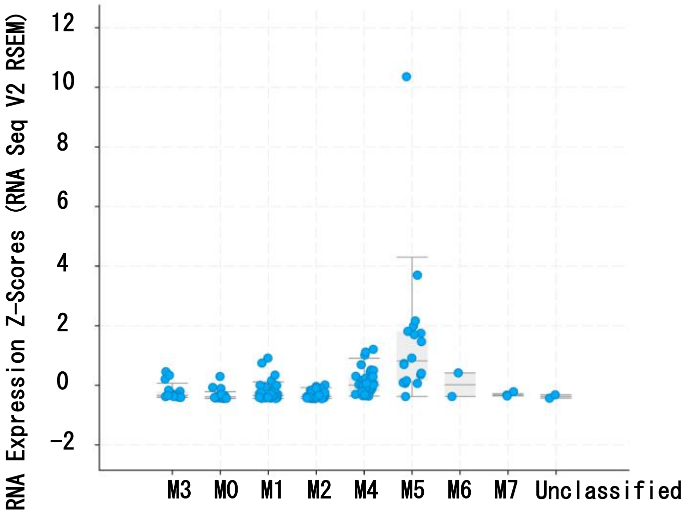

Figure S1. RNA-Seq data for *ITGA7* obtained from TCGA. The database (www.cbioportal.org) was accessed on 5 July 2019. Gene expression of *ITGA7* is shown based on FAB classification for each AML case and sample.

Gene/locus		Fragments per kilobase of exon per million reads mapped (FPKM)													
		AML M5 with GS at skin #1	AML M2 with GS at lymph node	AML M4 with GS at CNS	ABL with GS at lymph node	AML M2 with GS at vagina	AML M5 with GS at skin #2	AML M1 with GS at thymus	AML M2 #3 (control)	AML M2 #5 (control)	AML M2 #6 (control)	AML M2 #7 (control)	AML M2 #8 (control)	AML M2 #9 (control)	AML M2 #10 (control)
ITGA7	chr12:56078353-56106089	12.7666	0.478732	4.14507	4.29757	2.14742	11.0322	0.028828	0.368422	1.75072	0.293124	0.131476	0.769194	0.260048	0.422837
ITGB1	chr10:3189245-33247293	14.8676	3.80194	44.2208	23.1991	31.1281	29.2769	4.65319	3.88173	39.5121	63.2001	11.3681	2.92001	5.01898	1.86368

Figure S2. RNA-Seq data from this experiment. FPKM of *ITGA7* and *ITGB1* are shown.

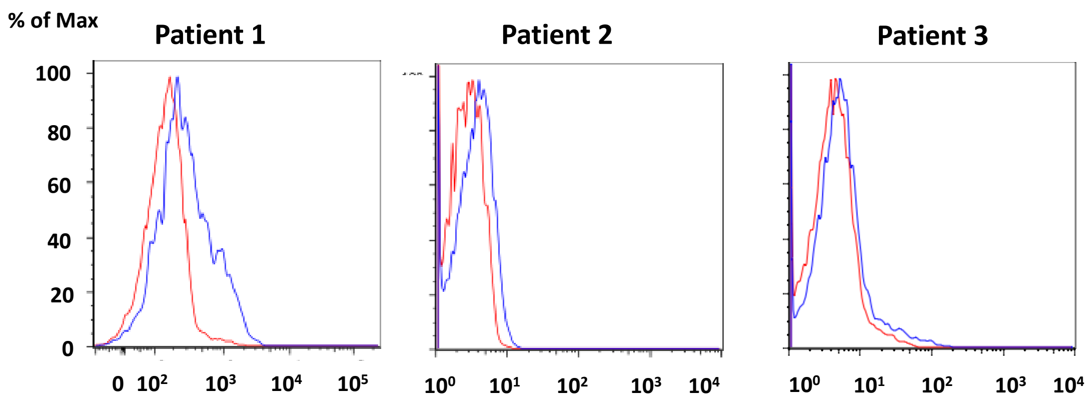

Figure S3. Flow cytometric analysis of bone marrow specimens. Anti-integrin $\alpha 7$ (blue line) and isotype control (red line) curves are shown. The vertical axis represents the percentage of cells. The horizontal axis represents fluorescence intensity of FITC.

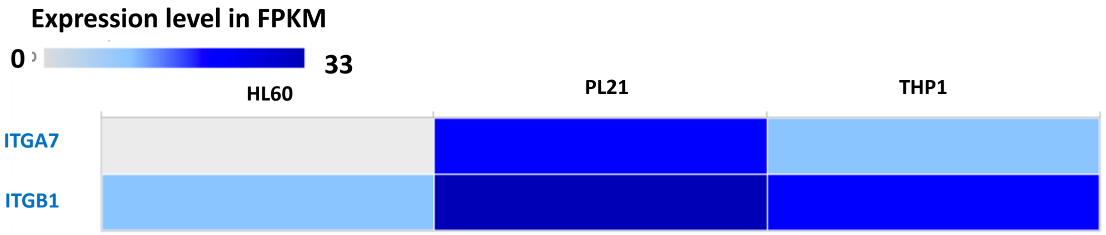

Figure S4. RNA-Seq data of human cell lines from Genentech via the Expression Atlas. The expression level of each gene and cell line is indicated by the intensity of the blue color, and the relationship between concentration and FPKM is shown in the figure. The Atlas (https://www.ebi.ac.uk/gxa/home) was accessed on 21 December 2018.

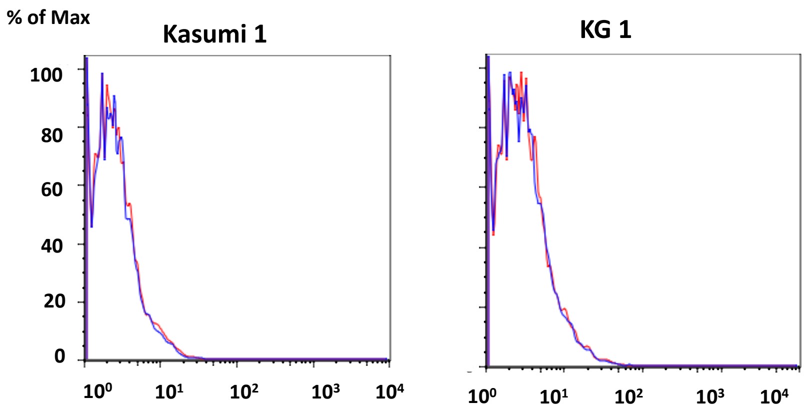

Figure S5. Flow cytometric analysis of AML cell lines. Anti-integrin $\alpha 7$ (blue line) and isotype control (red line) curves are shown. The y-axis represents the cell percentage. The x-axis represents fluorescence intensity of FITC.

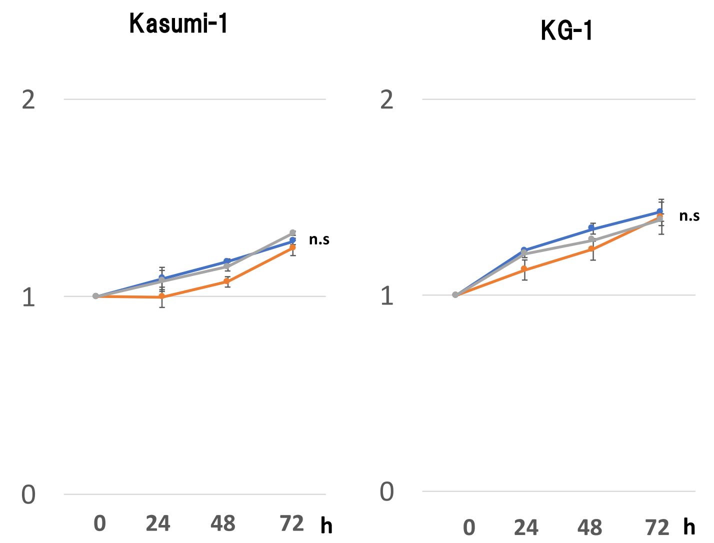

Figure S6. Proliferation of AML cell lines with laminin isoforms. The y-axis represents the proliferation rate starting at 0 h. The x-axis represents various time points. Experiments were performed in triplicate. The lines indicate proliferation on dishes coated with laminin 211 (blue), laminin 411 (orange), or the uncoated control (gray) for Kasumi-1 (laminin 211 vs. laminin 411: $p=1.00$; laminin 211 vs. control: $p = 0.22$) and KG-1 (laminin 211 vs. laminin 411: $p = 1.00$; laminin 211 vs. control: $p = 1.00$) cell lines. The error bars represent standard error of the mean. $^*p < 0.05$ was considered statistically significant.

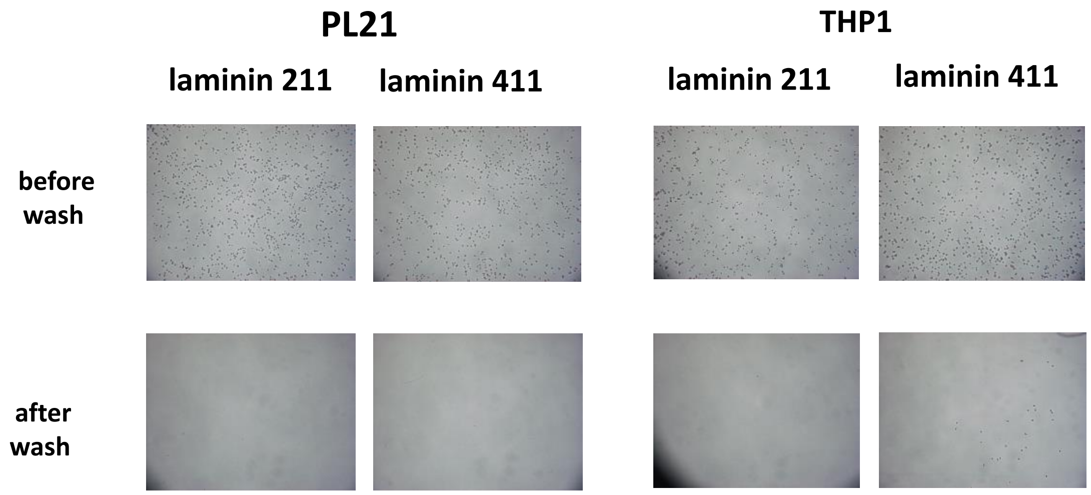

Figure S7. Adhesion assay. PL21 and THP1 were plated on laminin-coated 96-well plates at 1.5×10^4 cells/well and incubated for 1 h at 37 °C with 5% CO_2 and suspended in a plate shaker for 3 min. Thereafter, the cells were gently washed thrice with RPMI 1640 medium, and those remaining on the bottom were imaged.

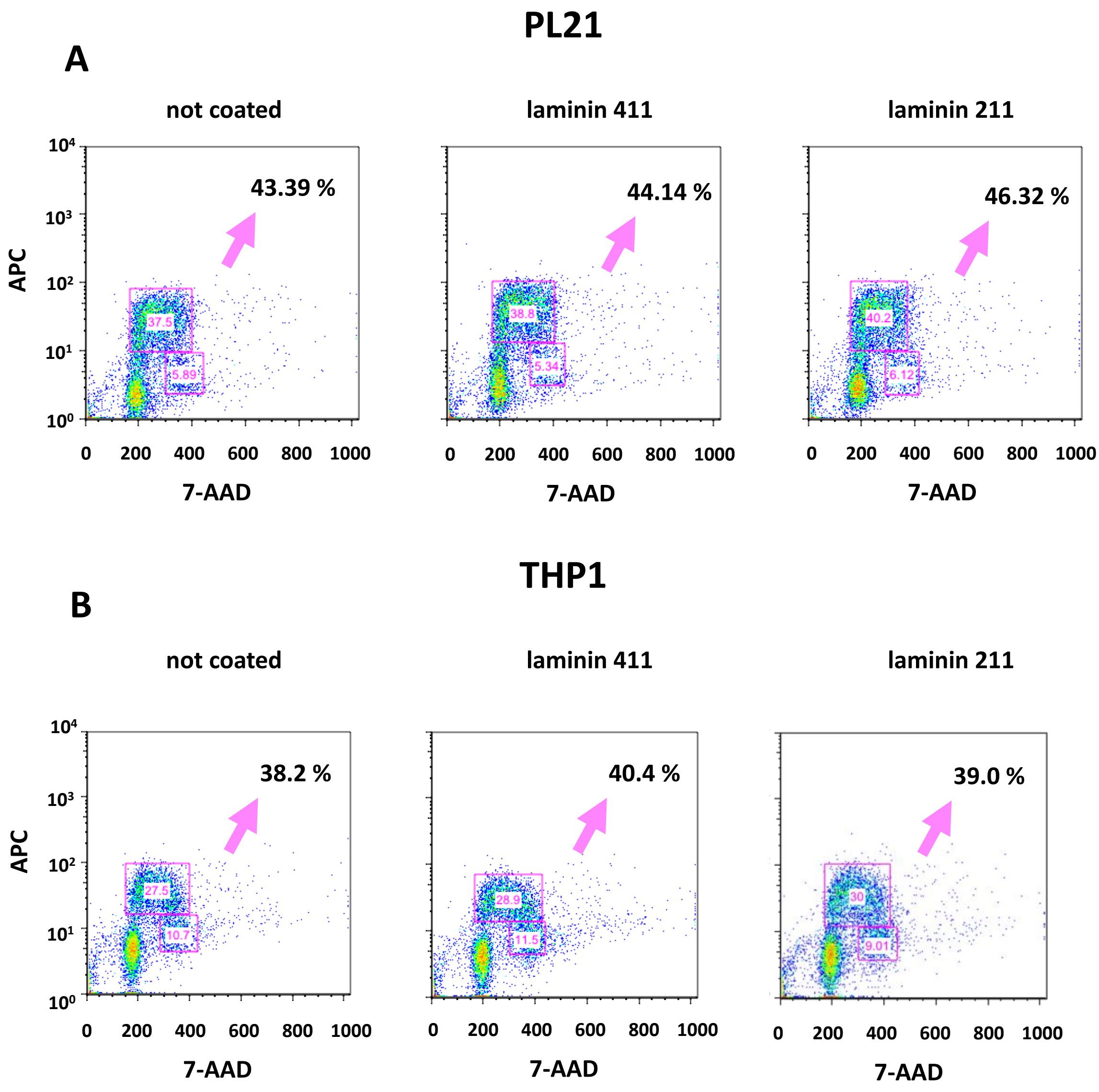

Figure S8. Cell cycle analysis via BrdU and 7-AAD staining. S phase and G2/M phase in (**a**) PL21 and (**b**) THP1 cells are shown within gates on the graphs. The y-axis represents APC fluorescent intensity. The x-axis represents 7-ADD fluorescent intensity.

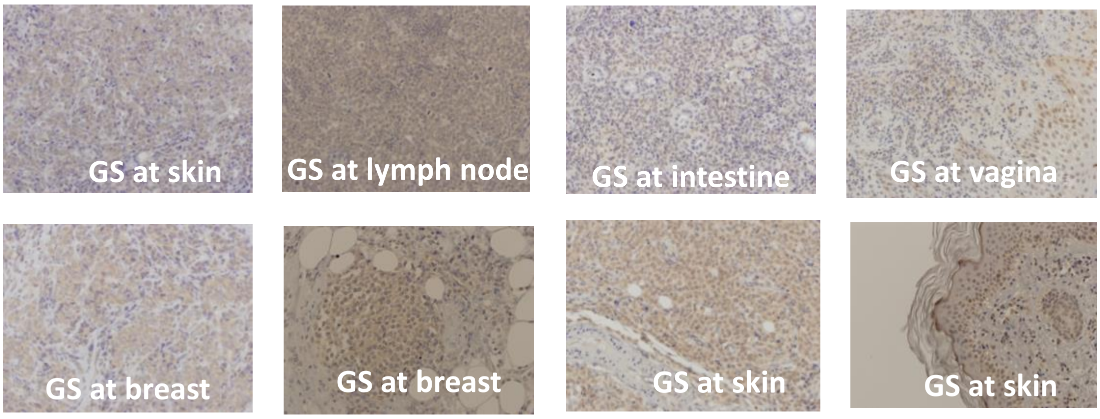

Figure S9. Immunohistochemical staining for laminin α1 subunit. Laminin α2 subunit stained positive in the ECM of GS sections.

Table S1. Background of the patients participating in the RNA-Seq analysis.

		With GS	Without GS
Number of patients		7	7
FAB classification	M0	-	-
	M1	1	-
	M2	2	7
	M3	-	-
	M4	1	-
	M5	2	-
	M6	-	-
	M7	-	-
	Multilineage dysplasia (MRC)	-	-
WHO classification	Acute basophilic leukemia (ABL)	1	
Site of GS	Lymph node	2	-
	Skin	2	-
	Vagina	1	-
	Central nervous system	1	-
	Thymus	1	-

© 2020 by the authors. Licensee MDPI, Basel, Switzerland. This article is an open access article distributed under the terms and conditions of the Creative Commons Attribution (CC BY) license (http://creativecommons.org/licenses/by/4.0/).
